# Faster but Less Careful Prehension in Presence of High, Rather than Low, Social Status Attendees

**DOI:** 10.1371/journal.pone.0158095

**Published:** 2016-06-28

**Authors:** Carlo Fantoni, Sara Rigutti, Valentina Piccoli, Elena Sommacal, Andrea Carnaghi

**Affiliations:** Department of Life Sciences, Psychology Unit "Gaetano Kanizsa", University of Trieste, Via Weiss 21, 34128, Trieste, Italy; University of Udine, ITALY

## Abstract

Ample evidence attests that social intention, elicited through gestures explicitly signaling a request of communicative intention, affects the patterning of hand movement kinematics. The current study goes beyond the effect of social intention and addresses whether the same action of reaching to grasp an object for placing it in an end target position within or without a monitoring attendee’s peripersonal space, can be moulded by pure social factors in general, and by social facilitation in particular. A motion tracking system (Optotrak Certus) was used to record motor acts. We carefully avoided the usage of communicative intention by keeping constant both the visual information and the positional uncertainty of the end target position, while we systematically varied the social status of the attendee (a high, or a low social status) in separated blocks. Only thirty acts performed in the presence of a different social status attendee, revealed a significant change of kinematic parameterization of hand movement, independently of the attendee's distance. The amplitude of peak velocity reached by the hand during the reach-to-grasp and the lift-to-place phase of the movement was larger in the high rather than in the low social status condition. By contrast, the deceleration time of the reach-to-grasp phase and the maximum grasp aperture was smaller in the high rather than in the low social status condition. These results indicated that the hand movement was faster but less carefully shaped in presence of a high, but not of a low social status attendee. This kinematic patterning suggests that being monitored by a high rather than a low social status attendee might lead participants to experience evaluation apprehension that informs the control of motor execution. Motor execution would rely more on feedforward motor control in the presence of a high social status human attendee, vs. feedback motor control, in the presence of a low social status attendee.

## 1 Introduction

**Fig 1:**
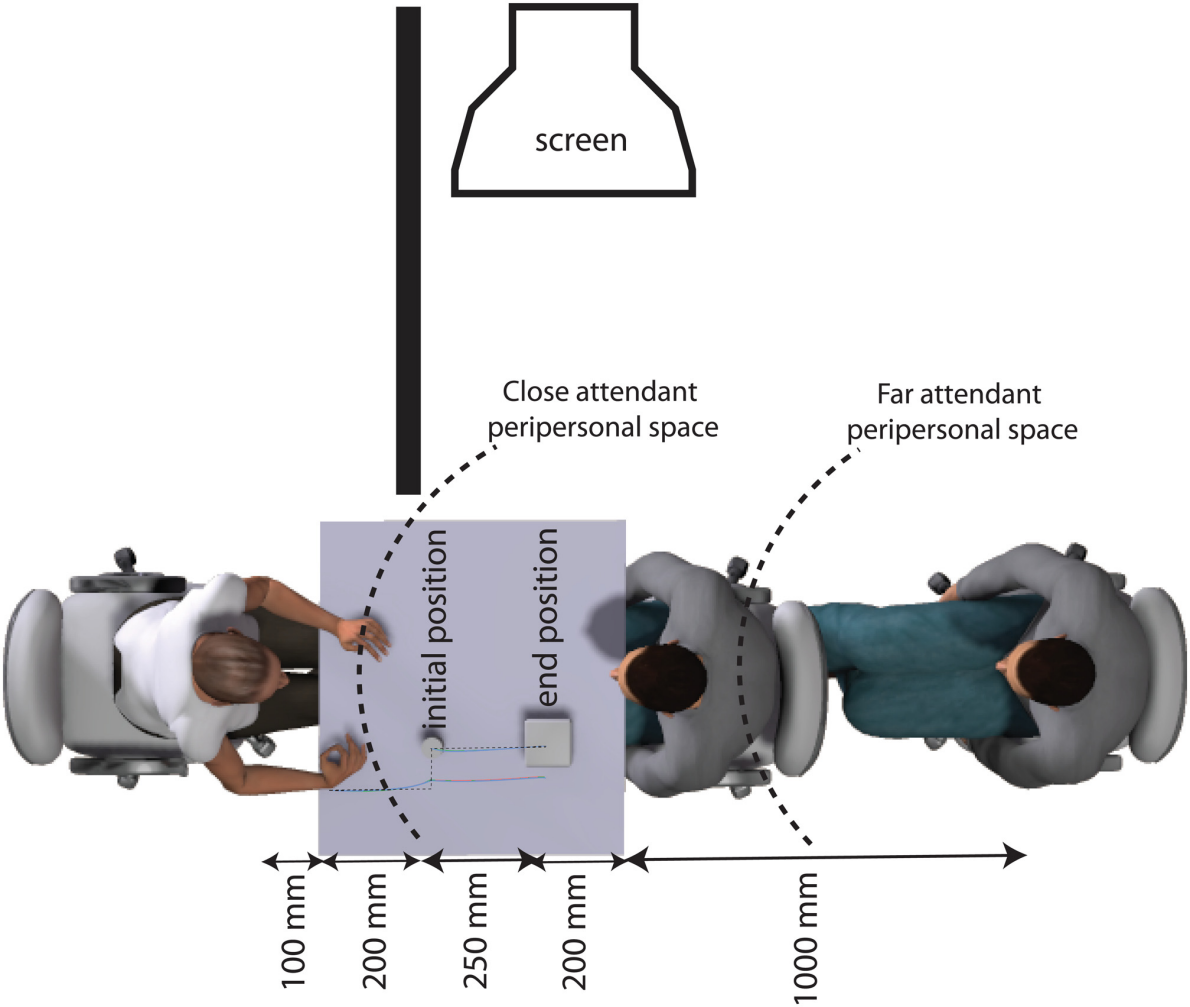
Experimental setting. A schematic of the bird's-eye view of experimental setting, with superposed the average trajectories (for the high and low social status conditions and the familiarization block), and the minimal path solution (dashed line) composed by connecting two straight segments both orthogonal to the working surface: one spanning from the IRED of the wrist in starting position to the IRED of the wrist in initial position, the other spanning from the IRED of the object in initial position to the IRED of the object in end position. The initial and the end target position as well as the respective distances from the starting agent's hand position are illustrated. The attendee is shown in the two tested conditions of distance (close and far) together with the distance of the far condition from the workspace (i.e. 1000 mm), and the corresponding peripersonal spaces (dotted semicircles).

**Fig 2:**
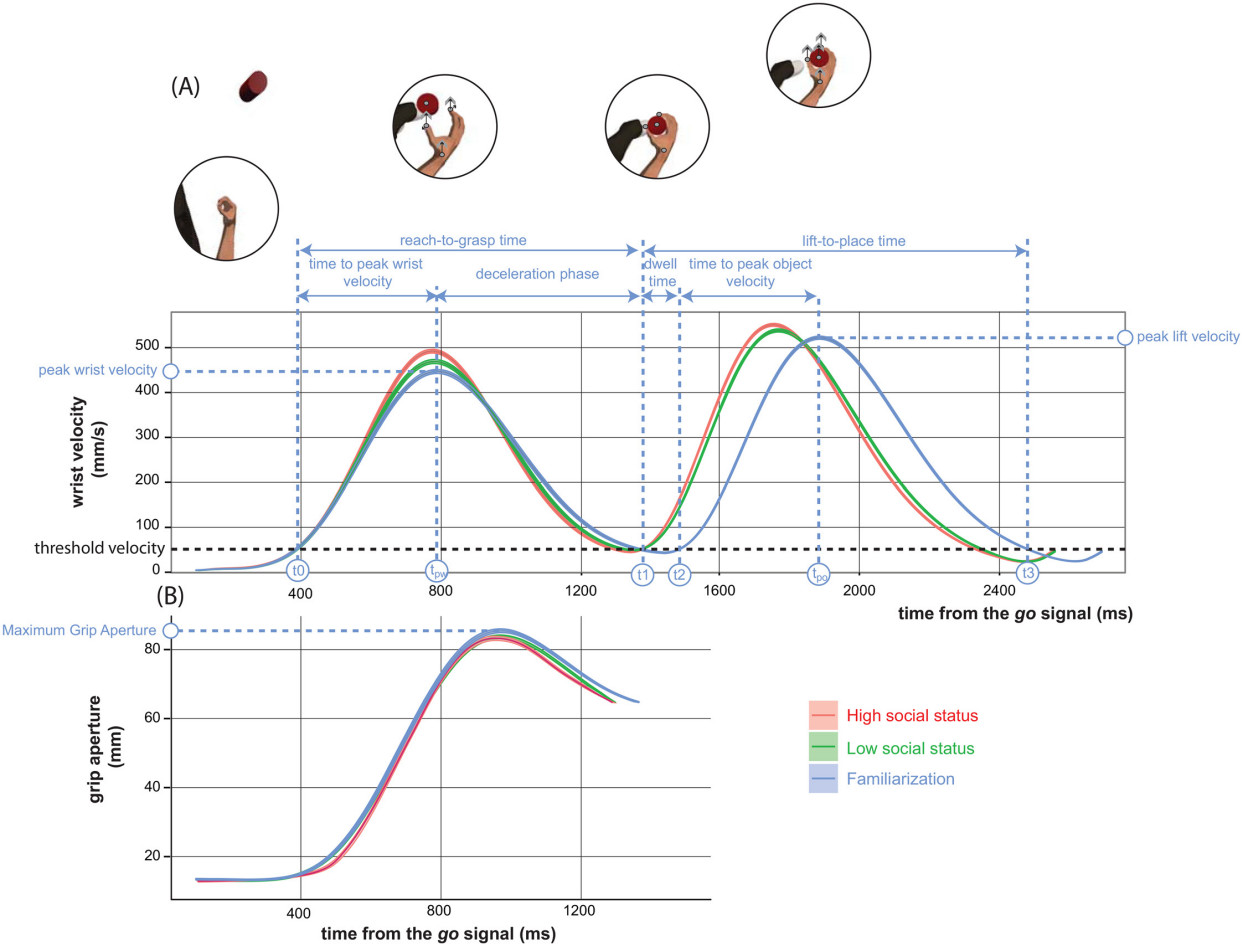
Relevant kinematic profiles for the hand movements performed by the agent in our task. The colour codes for the block (legend): high (red) social status, low (green) social status human and familiarization (blue). In A the average wrist velocity profile: shaded colored regions represent ± 1 standard error of the mean. The overall movement is subdivided into two phases: a reach-to-grasp phase from the time elapsed when the wrist marker exceeded the threshold velocity after the go-signal (t0) and the time the wrist marker dropped below the threshold velocity (t1); a lift-to-place phase from t1 to the time the object marker dropped below the threshold velocity (t3). The lift-to-place phase of the movement included a stop period occurring after contact of fingers with the object with the wrist IRED remaining below threshold velocity for a certain dwell time until t2. The icons above panel A provide a schematic representation of the shaping of the hand relative to the target object in the different phases of the hand movement ordered as follows: start position → reach-to-grasp phase with the wrist IRED reaching peak velocity (peak wrist velocity) → stop period → lift-to-place phase with the object IRED reaching peak velocity (peak lift velocity). Grey arrows represent velocity vectors as registered by the IREDs in real time for the encoding of the index, thumb, wrist and object 3D position over time, during the execution of the movement. Panel in B depicts the profile for the grip aperture, as occurring during the reach-to-grasp phase of the movement: shaded coloured regions represent ± 1 standard error of the mean. The maximum positive value along this curve corresponds to the MGA. In both panels A and B, the blue curve is a reference for evaluating the biasing effect of social status (red and green curves) relative to a condition with a lower level of familiarization with the motor task. In particular, the motor performance during the familiarization phase (blue curve) which relies on a significant degree on online feedback control (being the agent poorly familiarized with the task), differs to a greater extent from the motor performance in presence of the high (which relies on a significant degree on planning—red curves), than the low (as relying less on planning—green curves) social attendee.

Even the simplest prehensile movement is cast in a social context, and includes a complex sequence of goal-directed actions requiring perceptual and motor skills. Here we consider the act of moving an object toward a person/attendee (see Figs 1 and 2 for exemplar kinematics). This act subsumes the encoding of: a target object in terms of affordances (i.e. the types and motor patterns of interaction with an object), and of its extrinsic (i.e. distance), and intrinsic (i.e. shape and texture) properties, of the surrounding space (i.e. obstacles), and of the social context (e.g. the proximity of the target person and intentions), for a review see [1, 2]. Building upon models of action in which motor behaviours occur along a continuum of control regulated by interdependent feedback and feedforward modelling [3–5], this pattern of information sustains two components of action:

1. the planned component, relying on feedforward mechanisms based on the selection of adaptive motor programs given the environment, the goals of the actor and his/her internal states;
2. the on-line control component, relying on feedback mechanisms based on visuo-spatial characteristic of the target and actors. This component adds benefit of monitoring and occasionally adjusts motor programs in flight.

Actions relying more heavily on feedforward mechanisms are executed rapidly, as there is no need to account for the delay of feedback loops. Actions relying more heavily on feedback mechanism are executed slowly, and carefully, as they involve a continuous updating of the ongoing movement using information from sensory receptors [6–8], see [9] for a review.

However, when executing motor acts, people are almost blind to the number of possible motor solutions to perform. So far, the selection of motor solutions has been shown to be deeply rooted in visual information conveyed by the target object [1, 10–16]. With this regards a large body of research has focused on how hand movement kinematics are shaped by the processing of visual information and on their interaction with primitives, schemata and other types of spatial features of objects, in conditions in which the agent performing the action is in isolation. These strands of research are in line with paradigms suited to investigate individual minds in isolation, e.g. see the visuo-motor channels theory [17, 18]; but see also [19, 20].

Developing upon this approach, recent studies have investigated the effect of a new type of visual information about social context: communicative gestures, as elicited visually through gestures signalling a request of communicative intention, i.e. a social intention [21–31]. Becchio et al. [21], but also [30], asked participants to reach towards an object, grasp the object (reach-to-grasp phase of the movement), and displace it from one spatial location to another (lift-to-place phase of the movement). In the single-agent condition participants acted in isolation, while in the social/non-social conditions participants passed the object from the same spatial location into a human partner's hand signalling/not-signalling her willingness to interact with her by opening her hand. The results showed a slowdown of the hand movement in the social condition compared to the single-agent condition and the non-social condition during both the reach-to-grasp and the lift-to-place phases of the movement. Such a difference has been interpreted as evidence in favour of a motor program relying more on feedback control in which the accuracy, rather than the speed of execution, is favoured when passing the object to another person, thus producing a more careful modulation of hand shaping [21]. Similar results have been reported by [23] who found a variation in the kinematics of the actions when placing a piece of food into the mouth of another person, compared to acts ending into a mouth-like aperture on a dummy head. Quesque et al. [24] relied on a similar paradigm and demonstrated the effects of peripersonal space on hand movement kinematics performed with social intentions (but see also [26] for a similar effect in the presence of a familiar but not of an unfamiliar attendee): a hand movement took a longer time to be initiated when intended to place an object within the peripersonal-action (at reachability-distance), but not into a farther, space of a human attendee. In a similar vein, [31] found shorter hand movement duration in a competitive rather than a cooperative social context.

This strand of studies suggests that humans have developed specialized process of attuning to the presence of other individuals, and to the information most useful for social interaction (i.e. their interaction space). This is further testified by studies showing that humans have developed a specialized ability to read the social intentions from the observed actions [32]. For instance, [33] have shown that 2-day-old newborns were able to discriminate between visual cues indicating goal-oriented and non-goal-directed actions. Similarly, [34] demonstrated that adult individuals were able to appraise a performer’s intentions (i.e. competitive vs. cooperative) by simply looking at performers’ kinematics.

Together, these results are in line with the idea at the basis of the motor simulation theory [35–38]: motor processes underlie not only the execution of actions but also the understanding of other people’s intended action. Action is afforded by the observation of others’ communicative gestures activating the same feedforward mechanisms used for controlling individual's own actions [4], and enabling the agent to monitor their movements [39, 40].

However, in ordinary conditions, social factors influence our behaviour purely, in the absence of explicit visual signals like communicative gestures: for instance, on the basis of the knowledge of the social status of other persons [41–44], which is the focus of the current study. Though social status is an essential and pervasive component of everyday life, studies on its pure effect on the kinematic parameterization of prehensile movements are lacking. Crucially, we interact with others in a social context, in which our behavior is influenced by social norms, categorizations and stereotypes, with motor and affective cortex excitability based on simulative neural responses modulated by the perception of others’ status, group membership and similarity [45–52]. Our aim is to investigate whether the kinematics of a grasping-and-lifting-to-place action is modulated by a passive attendee’s social status.

### 1.1 Why do we need to study the relation between social status and hand movement kinematics?

Two series of findings on the effects of social status on different aspects of motor behaviour attest that it should be worth exploring its role in the particular case of hand movement kinematics.

Firstly, gaze allocation has been shown to powerfully affect the coordination of hand movements and to depend on social status [53–56]. [57] found that high status individuals are gazed at much more often, and for longer, than low status individuals, even over short, 20-s videos. Other studies attested that the eyes are a powerful attractor for overt attention in both schematic [58], and complex natural scenes [59]. The fact that social status deeply shapes gaze allocation has been further demonstrated by [60, 61]. Authors found a stronger gaze-cuing effect for high status faces than for low status faces, independent of the specific identity of the face. The link between gaze allocation, social context and action is clarified in a recent study of [62]. In particular, in Experiment 3, [62] asked the participant to reach, grasp and lift a bottle in presence of an empty glass, while the experimenter was facing the participant. The gazing behaviour of the experimenter was manipulated in order to allow or not the establishment of eye-contact during the motor act. The experimenter looked at the participant in the direct gaze condition, adopting a neutral body posture—see the right picture of Fig 1 Experiment 3 in [62]—or directed her gaze down, in the not directed gaze condition, adopting a contracted body posture—see the left picture of Fig 1 Experiment 3 in [62]. Eye-contact produced a faster grasp movement, as if the direct gaze together with the neutral (not contracted) body posture was implicitly interpreted by the agent as a request to execute the task quickly. Thus, inferred social status of others may be mapped onto our own sensorimotor system modulating our motor behaviour in a consistent manner. Notably, the faster motor execution found by Innocenti et al. [62] in the direct *vs*. not direct conspecific's gaze condition is in contrast with the slow-down of the hand movement observed when an explicit requirements of social interactions between the agent and the attendee is observed vs. not observed during the motor task [21–30]. This suggests that social context might affect in different ways the trade-off between the speed of motor execution and the carefulness in motor execution.

Secondly, the selection of motor responses has been shown to largely depend on social facilitation, i.e. the improvement in performance produced by the presence of others [63, 64]. For instance, [64] found that young individuals could spin fishing reels more quickly when performing in pairs than when alone. This effect is in line with the social arousal account to social facilitation, which has been originally put forward by Zajonc's [44]: the minimal condition that triggers social facilitation effects is the mere presence of a human audience. Importantly, this account suggests that the occurrence of social facilitation is independent of the social features of the attendee, such as her social status or her monitoring capacity. Indeed, any human audience is likely to elicit a status of alertness in the performer. Therefore, the performer seeks to respond to the potential actions of the attendee, which produces an improvement in the performance on simple/consolidated tasks. Cottrel [65] further developed the initial Zajonc's claim [44] and suggested that the social facilitation effect is dependent on social features of the attendee. According to such a view, social facilitation does occur when the observer endorses a role of monitoring the agent's performance, and induces a sense of evaluation apprehension in the agent, i.e. evaluation apprehension account to social facilitation. In such a view, changes in behavior that result from the presence of others depends on individual’s knowledge of whether the presence of others in a given situation is related to aversive or rewarding outcomes, rather than on social arousal per se. The evaluation apprehension account to social facilitation was supported by numerous studies showing that social facilitation occurred especially when the attendee was able to evaluate the actor’s performance (e.g. [66–68]). The presence of an attendee, who allegedly may evaluate the individual's performance, elicits a state of evaluative apprehension that affects the individual's performance in a way consistent with social facilitation [69]. As a case in point, several studies (see [70] for a meta-analysis) demonstrated that the presence of high status and expert individuals, compared to equal-status individuals or peers as the audience, caused more facilitation of simple/consolidated task's performance.

Notably, the effect of monitoring on grasping behaviour has recently been investigated by [71] in dyadic interactions in which task requirements implied an asymmetric role assignment with participants acting either as followers or as leaders, see [72] for a review. Importantly, even when not explicitly instructed, leaders increased the "communicative" properties of the act providing implicit cues regarding the action to be jointly performed: they performed movements selectively emphasizing kinematic parameters while reducing movement variability. The different pattern of motor behaviour found in leaders and follower by [71] is consistent with recent evidence suggesting a common anatomical substrate in the ventral striatum for the encoding of social factors relevant to interaction and reward processing [30, 73]. [30], indeed, used a motor task requiring agents to reach, grasp and lift an object towards another person with or without social intentionality and demonstrated that only those Parkinson's disease patients receiving a dopaminergic therapy necessary to sustain the basal ganglia (the main recipient of ventral striatum output), produced a pattern of kinematic responses similar to normal participants.

One possible interpretation of these results is that some social effects on motor control might be based on evaluation apprehension as mediated by the reward network. Evaluation apprehension might trigger agents to create forward models of their own motor behaviour flexibly adapted to the knowledge of others monitoring capacity. On such a basis, we hypothesized and tested whether, consistently with an evaluation apprehension (but not a social arousal) account to social facilitation, visually-guided motor control is affected by the social status (low vs. high) of an attendee monitoring an agent’s prehensile movement. Indeed, compared to low status, high social status individuals are perceived to be more competent [74], and they typically endorse roles that require the monitoring and the evaluation of others [75].

### 1.2 Rationale and Expectations

In order to test the above-mentioned hypothesis we selected: (1) only male agents; (2) two similarly looking male actors (A and B) attending the agent motor task endorsing an high status role and a low status role; (3) two pairs of curriculum vitae (CVs) that described a male individual endorsing a high status role and a low status role, that according to the results of a Pretest (available as a supplementary file, S1 Document) were reflected by well distinct subjective ratings of social status and income, but similar ratings of valence (for a similar procedure, see [74, 76]).

Note that, the assessment of prestige and economic success of a given target, operationalizes the perceived social status. This measure has been found to be predictive of the ascription to a target of social status related attributes, such as competence, agency, and power in different cultural contexts, for a review see [77]. Furthermore, previous studies have relied on a similar manipulation of the social status of individuals via fictive CVs associated with each person, and they have found that the different social status of distinct individuals, acquired through episodic learning, was effective in shaping spontaneous cognitive processes, such those involved in the gaze-cuing effect [61].

We decided to restrict our study to the only male participants and male attendee gender congruent condition (not including the corresponding agent/attendee gender congruent condition with woman as agents, and the 2 corresponding agent/gender incongruent conditions) so to maximize the effectiveness of our social status manipulation. Such a choice, although limiting the external validity of our study maximizes its internal validity being motivated by several studies rooted in both the evolutionary psychology and social cognition traditions [78–94] (see the supplementary file S2 Document for further details).

We tested participants individually in two successive motor tasks (counterbalanced across participants) distinguished by actors endorsing roles with opposite status (high vs. low). The motor task was to reach towards an object, grasp the object, and displace it from one spatial location to a straight-ahead target spatial location, in presence of a passive human attendee (high or low depending on the status condition). This motor task controlled for the effect of end-goal accuracy given that the agent was always asked to place the same object in the exact same physical end position.

The spatial proximity of the attendee with the target spatial location was randomly varied across trials in a within subject fashion (see Fig 1). We carefully instructed the actor to move closer or farther from the agent depending on the distance condition (near vs. far). In line with Quesque et al.’s ([24]; but see also [26]) this manipulation of the distance of the attendee was intended to control for residual effects of communicative intentions, while keeping constant the status of the attendee and the arousing effects of interpersonal-social distance. Varying the distance of the attendee from the target spatial location, we indeed defined two different types of potential interaction between the agent and the attendee. Following [95], in our setting (see dotted semicircles in Fig 1), when the attendee was in the near distance condition, the object was leaned inside the attendee's peripersonal space (being 20 cm far from the attendee). When the attendee was in the far distance condition, instead, the object was leaned outside the attendee's peripersonal space (being 120 cm far from the attendee). Both distance conditions however were presumably comparable in term of the arousing effects that might rise from the comfort/discomfort of the interpersonal-social distance between the agent and the attendee. The attendee-to-agent distance was indeed always well above 600 mm (750 mm in the near and 1750 mm in the far distance condition), that according to the results of [95], is the average value below which a males judge his distance from a confederate (in either a virtual or real setting) to be comfortable (interpersonal-social distance). As a consequence, in our design, any significant effect of the attendee distance should be interpreted as a by-product of communicative intentions, not of social arousal. The spatial precondition for the occurrence of a social interaction was indeed met in the near (the performer is acting within the attendee's peripersonal space), not in the far distance condition (the performer is acting outside the attendee's peripersonal space). In both cases the attendee-to-agent distance was always above the interpersonal-social (comfort) distance, thus arousing the performer by a similar extent.

In the present study, we manipulated both the social status and the attendee's distance as a within-subjects factor, and the status ordering as a balancing between-subjects variable. This crossover design was needed to avoid a possible carryover effect intrinsic to an experimental design in which the same agent/participant performs actions monitored by attendees with opposite status in successive sessions.

Before entering the two social blocks every participants, after a motor training, performed the exact same motor task in presence of a dummy, rather than a real human person (familiarization phase). Such a methodological choice is inspired on computational models that have suggested that the ability to adapt to others’ behaviour during social interactions might rely on the same feedforward mechanisms supporting motor learning [4, 96, 97]. On such a basis, we treated the familiarization phase as a warm-up session that allowed participants to achieve a similar level of dexterity with the motor task before entering the two social blocks. The familiarization phase controlled for the effect of salient human-like visual information on the planning and execution of action (a dummy human body-shape being placed in front of the agents with simulated eyes for the establishment of eye-contact), but not for the effect of motor learning.

Hand movement kinematics collected during the familiarization phase were thus meant to provide a reference measure of the agent's motor act for qualitatively evaluating the biasing effects of social status on the planned-control component of action. During the course of skill acquisition, motor learning is known to affect action by changing movement control from feedback to feedforward, with movements that become faster as a function of practice [98–101]. Accordingly, we expected the familiarization performance to rely less on feedforward mechanisms than any one performance in the two social blocks, given that the agent was less familiarized with the task. This should produce slower hand movements with a more careful shaping of the hand.

Notably, relative to previous works on action and social context [21–31] the following additional features distinguished our setting:

1. The attendee was not involved at all in the execution of the action. He only watched the action without performing any gesture that might implicitly or explicitly be encoded by the agent as communicative;
2. The attendee was instructed to monitor the action performed by the agent, thus implicitly increasing the perceived surveillance by the agent;
3. The attendee was external to the aim of the experiment (being an actor rather than the experimenter) and unaware of the experimental hypotheses in order to minimize the possible effects of compliance on prehensile hand movements (but see also [25–27] in which the attendees were naïve participants);
4. The social status of the attendee was explicitly manipulated providing the participants with two ostensibly CVs, which differed only in terms of occupational roles, namely a high vs. a low status job.

### 1.3 Which expected direction of the social status effects?

We measured several kinematic indices of prehensile movement proficiency sensitive to social context, at the two levels of attendee distance (close vs. far), for two social status of the attendee (high vs. low), to test the effect of a passive attendee’s social status on kinematics of a grasping-and-lifting-to-place action. In this section, we reported how the direction of this effect is expected to vary depending on alternative accounts to social facilitation.

Reframing reach-to-grasp into a collision avoidance task [102], rather than a simple type of targeting task [103], suggests possible different strategies for the control and execution of prehensile movements in the presence of a high social status (with high monitoring capacity) vs. a low social status attendee (with low monitoring capacity). A difference in the qualitative spatio-temporal structure of reach-to-grasp movements has been shown to occur as a function of the physical properties of objects [104], their texture [105], aging of the agent [106] as well as end goal accuracy [7 8, 107]. In particular, [104] suggested that object's affordance properties elicit either "a ‘stop’ motion, where the hand reaches the object then pauses to position the fingers", or "a ‘fly-through’ movement, where the hand reaches and grasps the object without a pause" ([106] p. 1–2). Interestingly, the safety margin of the reach-to-grasp task to hand size was found to be predictive of the spatial structure of the reach-to-grasp. For instance, older agents with reduced safety margins (due to a reduction in joint flexibility), showed a greater propensity for a ‘stop’ reach-to-grasp movement than younger agents: they performed the motor act more slowly and dwell on stop movements before lifting objects for longer times than younger agents.

Notably, these different spatio-temporal structures of reach-to-grasp movements reflect different contributions of the planned-control components on prehensile movements:

1. a "fly-through" movement [104] occurs when the grip safety margin is high, and there is a low need for online movement corrections. It relies more heavily on feedforward motor control mechanisms as being characterized by a fast movement;
2. a "stop" movement [104] occurs when the grip safety margin is low, and the execution of the motor act requires a continuous updating of the ongoing movement using information from sensory receptors. It relies more heavily on feedback motor control mechanisms as being characterized by a slow movement.

In a similar vein, here we expected that the presence of a high vs. low social status attendee might produce an analogous effect on the hand movements according to an evaluation apprehension, but not to an arousal based account of social facilitation. In particular, we considered two alternatives effects of evaluation apprehension on hand movement kinematics resulting from the speed/accuracy trade-off in prehension [9, 103, 108]: the faster the hand transport movement, the lower the carefulness of execution of the performed act (signalled by the deceleration phase of the hand movement, and the maximum grip aperture), and vice-versa. Specifically, the effect of evaluation apprehension on the selection of the optimal motor control model sustaining the execution of the prehensile act requested in our motor task might be finalized to the optimization of either the speed, or the carefulness of execution. We herewith refer to these two alternative expectations about how evaluation apprehension might affect the execution of the hand movement as:

- Expectation 1: if evaluation apprehension underpins the execution of a fast motor act;
- Expectation 2: if evaluation apprehension underpins the execution of a slow and careful motor act.

Notably, these alternatives expectations are consistent with seemingly contrasting results about the effect of social factors on hand movement kinematics: the faster hand movements produced by a competitive social context and the gazing of a conspecific found by [62] and [31] (consistent with Expectation 1), vs. the slow-down of hand movements produced by social intention and cooperation found by [21–30] and [31] (consistent with Expectation 2).

Let us consider Expectation 1 first. The agent in presence of an attendee with high monitoring capacity (high status condition) would create an optimal temporal feedforward control model of the motor act. Specifically, the hand transport movement would be planned so to be fast. This would minimize the likelihood that the attendee with high social status (but not low status) would detect unaware motor errors, while tolerating some small risks that the performed motor act would include execution inaccuracies (like slippages, inaccurate placement of target object). This control strategy would underpin an hand movement similar to the one named by [104] "fly-through", in which the speed of execution is favoured over the accuracy of execution in presence of a high (but not of a low) social status attendee. Specifically, a movement resulting from this type of motor control strategy (favouring the speed of execution over the carefulness of execution) was expected to be characterized by a short duration of the deceleration phase of the movement, a large amplitude of the peak wrist velocity of the hand with an early occurrence of the time at which the peak wrist velocity is reached, and a short duration of the finger-to-object contact phase before the lifting, as well as by a fast lifting of the object [14, 109, 110]. In addition, the deviation between the actual size of the object and the maximum grip aperture, being a kinematic index of motor proficiency [111–114], was expected to be small: substantiating a tendency towards a low careful modulation of hand pre-shaping. In presence of a low social status attendee, the agent would select a model of the hand transport movement relying more heavily on feedback on-line control: underpinning a less fast but more careful execution of the motor act than in presence of a high status individual.

The opposite expectation raises if evaluation apprehension induced by the high status attendee drives the creation of an optimal control model optimizing the carefulness in motor execution to the detriment of speed (consistent with Expectation 2). The motor act would be based on a motor control strategy similar to the one produced by social intention [21–30] and cooperation [31]. Specifically, the presence of a high status attendee, would promote a feedback (rather than a feedforward) movement control of the motor act minimizing the risks of execution inaccuracies (like slippages or misplacements of the target object), while tolerating a possible slow down of the transport hand movement (resulting from enhanced precision requirements). A longer execution time together with a longer deceleration period were indeed found when grasping a small and/or fragile object, when the task was complex [7, 8], or when the act was performed with a specific social intention e.g. [21], see [9, 26] for a review on motor and social intention, respectively. On such a basis, a lower execution speed in the high social status condition likely indicates that the agent is seeking to compute a more careful approach and placing of the target object in order to be positively evaluated by the high status attendee. By contrast, when the object had to be placed in front of an attendee with low monitoring capacity (i.e. low social status condition) the determination of the contact points for the fingers might not be so crucial, and the execution speed can be faster. A similar rationale applies for the grip aperture, which is known to increases as the biomechanical requests of the grip increase. A bigger grip aperture allows for a greater safety margin and it is adopted, for instance, when the object is fragile, slippery or complex to be managed. With this in mind, we could predict that the grip aperture will be larger in presence of a high social status attendee relative to a low social status attendee.

Note that according to a social arousal account of social facilitation any one of the two above described motor control strategies (one favouring the speed over the carefulness of execution or vice-versa) would be selected when acting in the presence of any types of human attendee (regardless from his social status). According to this view, the only presence of a human audience is enough to elicit a state of alertness in the performer: the entity of such alertness being independent on the status of the attendee [44]. Consequently, according to a social arousal account of social facilitation, no difference in the kinematic indices of prehensile movement proficiency was expected between the high and the low social status condition. This view thus provides a theoretical ground for a null hypothesis on the effect of the social status of the attendee on hand movements.

In sum, the present study seeks to answer the following question: are we more prone to act favouring speed over carefulness, when reaching, grasping and placing an object in presence of a high but not a low status attendee? If the social status of attendee drives the selection of one or the other motor strategy, then we expected to find effects of social status at the level of both the speed and accuracy of motor execution. These effects should provide an objective measure based on hand movement kinematics, rather than a subjective measure based on self-description, of the way social facilitation (and the internal states induced by it, like evaluation apprehension or social arousal) affects behavior. This is needed to avoid well-known problems related to the self-referential assessment of internal states; i.e. to "emotional self-awareness" [115, 116].

## 2 Method

### 2.1 Participants

Thirty-one right-handed male students of the University of Trieste, (*M* = 22.5, range 19–28), participated in the experiment. All had normal or corrected to normal vision, and were naïve regarding the purpose of the experiment (as confirmed by the result of post experimental questioning on compliance, see the Procedure section). Participants took part in the current study in exchange of course credit.

Participants were randomly assigned to one of the eight conditions resulting from the full factorial combination of our 3 balancing variables: Ordering of the social status block (high social status ⇒ low social status; low social status ⇒ high social status), Actor Role (Actor A → high social status & Actor B → low social status; Actor B → high social status & Actor A → low social status), Type of jobs (high social status → business consultant vs. low social status → temporary worker; high social status → entrepreneur vs. low social status → metalworker). Each condition included 4 participants, with the exception of the condition in which the actor A endorsed the high social status role as an entrepreneur and the first block was performed in presence of a low social status attendee including 3 participants.

Experiments were undertaken with the understanding of each participant, the approval of the Human Research Ethics Committee of the University of Trieste (approval number 52) in compliance with national legislation, the Ethical Code of the Italian Association of Psychology and the Code of Ethical Principles for Medical Research Involving Human Subjects of the World Medical Association (Declaration of Helsinki). All participants provided their written informed consent prior to inclusion in the study and therefore behaved as active participants in the entire data collection. Hand movements kinematics were filed in raw documents. Dataset is available as a supplementary data file (S1 Dataset)

### 2.2 Apparatus, Stimuli and design

As shown in Fig 1, the agent was seated in a brightly lit room on a height adjustable chair, with their sagittal body midline orthogonal to the working surface (i.e. a 75 × 75 table colored cyan). The attendee was placed on the opposite side of the workplace in a straight-ahead position relative to the participant, carefully adapting the height of the two individuals (i.e. the performer and the attendee) so that their eyes were exactly at the same eye height (47 cm from the workplace tabletop). Before each trial, the right hand of the participant rested at a starting position (on a black circular velvet cloth 3 cm) with the index finger and the thumb gently opposed. The target object was a mid grey wooden cylinder (ray = 3 cm; height = 8 cm; weight = 150 gr) placed above a circular velvet cloth of 3 cm at the beginning of each trial. It was located 20 cm away from the starting hand position along the line orthogonal to the working surface. The end position (i.e. the location on the work surface in which the target object was asked to be placed), was signalled by a black circular velvet cloth of 3 cm that was placed above a wooden mid-grey block, 8 cm tall and 25 cm from the initial cylinder position along the line orthogonal of the work surface.

To control for musculoskeletal tension, we carefully adjusted the horizontal position of the participant at the beginning of each trial, so that, independent of the arm length, the joint angle of the elbow was 90°. The distance of the attendee from the end position of the target object was randomly varied across trials with half of the trials involving an attendee which was close enough to the end position of the target object to include it within his peripersonal space (20 cm); and half of the trials involving a far attendee with a target object falling outside his peripersonal space (120 cm). These distances (close = 20 cm vs. far = 120 cm) resulted by keeping the attendee facing the agents, at a distance of 0 vs. 100 cm from the workspace respectively. Both actors were carefully instructed to vary their distance from the workspace on a trial by trial basis as follows: they were asked to carefully move the height adjustable sliding chair on which they sit across two predetermined positions according to a written instruction ("close"/"far") appearing on a monitor (not visible to the agent, see Fig 1 for monitor position). Specifically, the attendee moved from the close to the far position sliding the chair, while keeping the wheels within two parallel lines painted on the ground that were orthogonal and centered to the workplace, from a position in which his body was fully attached to the workspace to a position in which the back of the chair was fully attached to the back wall of the room (111 cm far from the workspace and parallel to it). This operation took approximately 5 s to be performed by our actors.

A custom C++ program controlled the randomization of distance conditions (within block), the emission of the go-signal (a tone -880 Hz/200 ms- lasting 300 ms occurring with a delay of 3 s from the experimenter key press), and head and hand movement kinematics recording. The head and hand movements were recorded using an Optotrak Certus motion tracker with one position sensor revealing the signal from infrared markers with sub-millimeter resolution (by Northern Digital Inc., Waterloo, Ontario, Canada) with a sampling frequency of 100 Hz (see [117] for technical details). The position and orientation of the participant's head were tracked with three infrared-emitting diodes (IREDs) arranged on the back of the head. The position of each digit was calculated relative to three markers affixed to a bent rod, which was fastened at the tip of the index finger and thumb. These markers were used to measure the grasp component of the action. Another marker was fixated to the ulnar styloid process to track the wrist position, as needed to measure the transport component of the action during the reach-to-grasp phase of the prehensile movement. One marker was attached to a thin and rigid metal antenna sticking out of the cylinder at about 8 cm. According to previous research [104–106] this marker was used to track the object’s position during the lift-to-place phase of the prehensile movement. All experimental sessions started with the calibration procedure (see [118] for technical details). We controlled movement execution and agent's position across and during trials from a remote station hidden to the agent by a black curtain placed behind the attendee. The remote station was used by the experimenter to on-line monitor reaching kinematics and head position during and across trials.

The experiment was a block design. All subjects (except one) performed three different subsequent blocks in order to measure the effect of social factors on prehensile movements: first a familiarization non-social block, and then two experimental social blocks. The two social blocks differed in terms of the social status of the attendee. The order of the social status of the attendee was counterbalanced across participants.

In the familiarization non-social block, participants acted in the presence of a human body-shape placed in front of the agents. We used a human body-shape similar to the one that [62] used to control for the effect of gazing on hand movements in the presence/absence of human attendees. It was a grey silhouette cut-out of a human like body, with its visible part resembling the head and the upper trunk of a human body (shoulder to tabletop distance = 270 mm). The head depicted the outlines of a schematic face (a 220 × 200 mm oval), with two black crosses (30 × 30 mm) at 470 mm from the tabletop used as schematic standardized male eyes. The center of the two crosses lied along a line parallel to the workspace with one crosses being 3.25 cm to the left and the other to the right of the sagittal head midline. The crosses served the purpose of reproducing similar gazing behaviour in the baseline and in the social conditions providing a human like reference for the establishment of eye-contact. We resonate that this manipulation might be important given that previous findings on both schematic [58] and complex natural scenes [59] showed that the eyes of others are salient features for overt attention, which in turn depend on social status [57]. During the familiarization phase, the distance of the human body-shape was manipulated in the exact same fashion as during the successive experimental social blocks. The human body-shape was attached on his base to a rigid stick, which passed through a rail attached to the floor allowing the experimenter to adjust its position in depth on a trial-by-trial basis according to the exact same distances used in the experimental social blocks. The experimenter manipulated the distance of the human body-shape from a position that was invisible to the participant: on his back behind a black curtain.

In the two experimental social blocks, the participant performed the same motor act previously performed in presence of the human body-shape. During the social blocks, the attendee was one human individual, with a high or low social status role. In order to do that, we recruited two male actors (i.e. actor A and actor B), with a similar physical structure (e.g. height, weight, heir and eye color), who played either the role of a high or a low social status attendee. The social status was attested using fictive CVs (see the supplementary file S1 Document for further details). Each CV was associated with the color photograph of the face of the actor that was assigned to the high and low social status role. Playing the high social status role, the actor was dressed in a blue and gray suit, while playing the low status role, he was wearing a grey t-shirt. Both actors wore the exact same dark grey trousers. The same outfit was also used for the photo added to the CVs. To avoid any potential confounding effects of the physical features of the actors, half of the subjects run the experiment with actor A endorsing the high social status role and actor B the low social status role, and vice-versa for the remaining half of the participants (we referred to this balancing variable as Actor Role). Furthermore, to prevent any impact of the type of the experimental material describing the status role, two pairs of high and low status roles were used in a balanced fashion in the current study (business consultant and temporary worker vs. entrepreneur and metalworker; we referred to this balancing variable as Type of job). The two pairs of high and low social status roles were selected on the basis of a pre-test. Specifically, in each pair, one social status role was perceived to have higher prestige and income, namely higher social status, than the other status role. More important, the roles in each pair were comparable in terms of valence. Results and details of the pre-test are available as a supplementary file (S1 Document).

At the end of the three action blocks, every participant was provided a response form containing 4 items. The first two items checked whether the participants correctly recalled the association given during the instruction between each attendee and each type of job. They were two forced choice questions ("which is the job of attendee A/B") with 4 alternatives (business consultant, temporary worker, entrepreneur and metalworker). The remaining 2 items were devoted to check whether the jobs used in our CVs to implement the social status manipulation were actually perceived by our participants as conveying higher prestige and income based on self-referential assessment. The two items were displayed as 7 equally spaced neighbouring blocks with the upper part of each block displaying a number increasing from -3 (left) to 3 (right) in one unit steps. The following verbal labels filled the bottom parts of the blocks: extreme left blocks "less relevant/prestigious"; central blocks "equally relevant/prestigious"; extreme right blocks "more relevant/prestigious". Above each item a question was displayed in the following form: Relative to job of attendee A/B (reference attendee), how much was relevant the job of attendee B/A?. In half of the questionnaires the reference attendee was the one endorsing the high social status role and vice versa for the remaining half. This manipulation was intended to control for possible effects of the spatial orientation of the rating scale.

### 2.3 Procedure

Each trial of the motor task was constituted by the following four phases: (1) The experimenter first verbally exhorted the participant to look into the eyes of the actor/dummy person in front of him (in social blocks the experimenter also stressed the profile of the actor by ending the gazing instruction repeating his name and his work); (2) after 3s from the ending of the verbal instruction on the establishment of eye contact a tone (880 Hz/200 ms), lasting 300 ms, was presented; (3) and the participant was requested to start the prehensile movements; (4) when the action was terminated (i.e. the cylinder was grasped, lifted and placed on the wooden block) the participant was asked to return the cylinder to its starting position using the opposite hand to the one they used to perform the prehensile movement (i.e. left hand) and the next trial was presented.

The procedure included the following sequence of events: (a) Instructions; (b) A motor training session containing a random sample of 10 unrestrained motor acts to be performed in complete isolation to get accustomed to the motor task; (c) a familiarization session where the same motor task was performed in presence of a human body-shape whose position in depth relative to the workspace was randomly varied on a trial by trial basis according to two distances (0 *vs*. 100 cm); (d) the presentation of the CVs of both the human attendee; (d-e) two successive experimental-social sessions distinguished by the social status of the human attendee monitoring the motor acts (high vs low) with a variable position in depth relative to the workspace as in (c); (f) post-experimental questionnaire and questioning.

For 28 out of the 31 participants, complete experiment included: (1) 30 familiarization prehensile-movements trials performed in presence of a human body-shape resulting from 2 Distances (close vs. far shape) × 15 repetitions, lasting about 8 minutes; and (2) 60 experimental prehensile movements trials resulting from the full factorial combination of 2 Social Status conditions (high vs. low) × 2 Distances (close vs. far attendee), lasting about 26 minutes (including the time for changing actor and for recalibration). Three participants out of 31, performed 10, not 15, repetitions per-condition (one for each Ordering of the social status block, Actor Role, and Type of jobs) and one of them (belonging to the group with Ordering of the social status block = high social status ⇒ low social status; Actor Role = actor A → high social status; and Type of jobs = high social status → business consultant) did not performed the familiarization block.

Written instructions informed participants that they would have been asked to reach, grasp, lift and place a cylinder in front of unfamiliar attendees. In the instruction it was stressed that the motor task required: (1) to establish an eye contact with the attendee before a go-signal; (2) to adopt the same gazing behavior in front of the human body-shape during the familiarization motor session by firmly fixating the crosses painted on the dummy head before the go-signal; (3) to initiate as soon as possible the hand movement after the go-signal; (4) to keep the trunk and the head the more stable as possible during and across trials. Furthermore, in the instruction it was explicitly stated that the two actors would have monitored the motor act. The instructions also included a cover story. As part of the cover story, participants were told that, as in ordinary life, people know some pieces of information about individuals and that these pieces of information were provided to them by reading the two short CVs of the persons they would have to place the cylinder in front. Also, as part of the cover story, participants were informed that, in the first part of the experiment, they would execute the task in front of a dummy person (i.e. familiarization block) to get more accustomed with the motor task.

**Fig 3:**
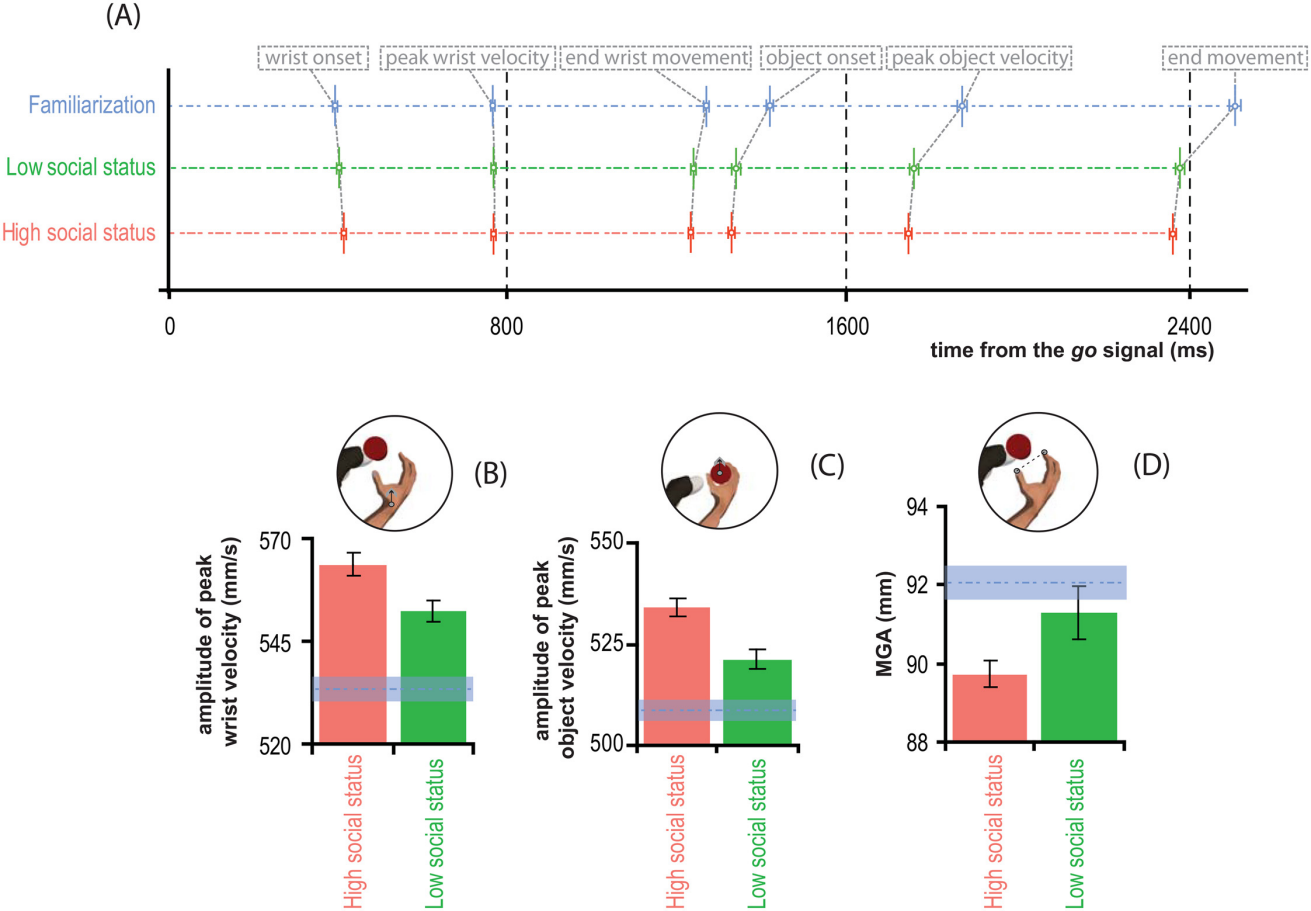
Kinematic parameters of prehensile movements are biased by the social status of the attendee. In A the average temporal parameters ± SEM, associated with the relevant events characterizing the motor act performed by the agent in our task are represented as staked segment for the two tested social status conditions (red segment for high social, green segment for low social) and for the familiarization phase (blue segment). The length of each segment is proportional to the time from the go-signal (ms). Each point along the segment represents a relevant kinematic event (see the topmost labels for the ordering and type of event). Each segment is subdivided by the points into six successive temporal intervals each representing the temporal duration of a relevant part of the motor act. A high social status attendee evoked the longest planning of the movement (longest first temporal interval), followed by the shortest execution of the act (all intervals successive to the first are considerably shorter for the high social status condition, relative to the low social status condition, and even more to the familiarization phase). (B, C,) Average spatio-temporal kinematic parameters ± SEM of the reach (B, amplitude of peak wrist velocity), and lifting (C, amplitude of peak object velocity) components of the movement, and a schematic view of the corresponding measure associated with each parameter (topmost insets). The panel in D shows the average spatial kinematic parameters ± SEM of the grasp (maximum grip aperture, MGA). In B, C and D horizontal blue lines represent the average values on the familiarization phase, ± SEM. These values are a reference for evaluating the biasing effects of social status relative to a condition with a lower level of familiarization with the motor task.

The actors/attendees were instructed to: (1) maintain a neutral expression and body posture during the entire session; (2) to change position in depth according to the distance condition (signaled on a screen on their right not visible to the agent as depicted in Fig 1); (3) to look at the participant’s eyes at the beginning of each motor trial; (4) and to carefully observe the agent prehensile movement by moving the gaze toward the hand of the participants as soon as the go-signal was delivered.

At the very end of the experiment, all participants completed the post-experimental questionnaire. Participants were first asked to recall the jobs of the two attendees by selecting from the first two items' questionnaire just one out of the four alternative jobs associated with each attendee. They were then asked to rate the differential prestige/relevance of each attendees' jobs by answering to the second pair of items. Finally, participants were screened for compliance through post-experimental questioning asking them: (1) whether in any time, during the two social blocks, they got the feeling to have acted applying different/similar motor strategies; and (2)—in the case they answer "different"—to describe the reasons at the basis of such a difference. Post-experimental questioning demonstrated that all participants except one (that was discarded from the sample) were unaware of the hypothesis of the study. All of them indeed reported that they were applying a similar action mode during the execution of the task in the two social blocks.

### 2.4 Data Analysis

A custom-made R software was used to analyze raw positional data offline. Individual data were interpolated through cubic spline smoothed and differentiated with a 2nd order Savitzky-Golay filter with a window size of 41 points. Closely following the procedure implemented by [119] we then used these filtered data to compute positional data, velocities and accelerations in 3D space for each fingertip, the wrist, and the object. The Euclidean distance between the fingertips of the thumb and the index finger (grip aperture) and the velocity and acceleration of the change in grip aperture was also computed. As in previous studies [15, 113] a wrist marker velocity raising above and falling below 50 mm/s was used as the threshold velocity defining the movement onset and movement end (Fig 2A). The criterion for onset and termination of the grasp closure was similarly addressed when the aperture open/closure rate of the fingers dropped above/below 50 mm/s respectively (Fig 2B). Following [104–106] we designed the instant in which the lift-to-place phase was initiated/terminated as the time in which the marker attached to the object exceeded/fell below a velocity of 50 mm/s. According to [104–106] the marker on the object, rather than the one on the wrist, was used to analyze the kinematics of the lift-to-place phase of the hand movement as the signal from the wrist marker was frequently missed when the hand get in contact with the object as due to occlusions.

As kinematic indices of prehensile movement proficiency we selected those that according to previous studies were most sensitive to differences in social attitude and high level object properties, e.g. [2, 31]. Specifically, we considered as relevant to test our experimental hypotheses the following variables obtained from wrist and object markers:

- Amplitude and time of peak velocity, related to the programming of the reaching act if computed on the wrist marker during the first reach-to-grasp phase of the action (Fig 2A, t_pw_), or to the programming of the lift-to-place act if computed on the object marker during the second phase of the movement (Fig 2A, t_po_)
- Deceleration phase of wrist and of the object, from time of peak velocity of the wrist (Fig 2A, t_pw_) to the end of the reaching (Fig 2A, t1) and from time of peak velocity of the object (Fig 2A, t_po_) to the end of the lifting (Fig 2A, t3). This kinematic has been shown to be a reliable measure of caution in the approach to the object (i.e. carefulness) being it dependent on the visual feedback concerning the ongoing reduction in relative distance (i.e. depth) between the moving hand and the target as controlled by the online programming component of the prehensile movement [120];
- Movement time, representing the total execution phase, from movement onset to end as subdivided into two subcomponents: one regarding the reach-to-grasp phase (from the onset of wrist movement to the onset of the movement of the object: in Fig 2A, from t0 to t1), and the other the lift-to-place phase (from the onset of the object moving to the end of the object motion: in Fig 2A, from t1 to t3) of the prehensile movement;

As a dependent measure of grasping performance, we considered the maximum grip aperture at hand preshaping. This was calculated as the maximum Euclidean distance between the tip of thumb and the tip of index finger as typically occurring within 70% of reach-to-grasp movement completion (Fig 2B). Such a measure was known to scale with: (a) the assessment of the object size-distance relations, e.g. [109]; (b) the transport component of hand movement, according to the speed/accuracy trade-off [103]; and (c) social factors [21, 31].

For the statistical analysis, we calculated separately, for all indices of prehensile movement proficiency, the individual values obtained for all trials under each of the two experimental block conditions and the familiarization phase (total trials collected = 2680). We thus discarded those trials that can not be interpolated smoothed and differentiated (220 from the reach-to-grasp phase and 42 from the lift-to-place phase). These generally corresponded to trials in which either the initial or the end of the grasping movement could not be identified correctly (the hand kept drifting), causing an occlusion of the IREDs for more than 20% of the total hand movement time. Furthermore, we removed from the analysis of both movement phases those trials collecting a time to movement onset smaller than 100 *ms* or larger than 900 *ms* (72). After the application of these exclusion criteria we removed those trials in which any one of the considered individual kinematics deviated more than 3 SD from the individual mean in each experimental condition (15 from trials of the reach-to-grasp phase; 21 from trials of the lift-to-place phase). Overall, we excluded from the analysis the 11.4% of the trials of the reach-to-grasp phase (98 from the high, 88 from the low, 123 from the familiarization block), and the 6.1% of trials of the lift-to-place phase (27 from the high, 18 from the low, 120 from the familiarization block).

Since the number of valid trials per Distance of the attendee by Social status condition varied between participants (*M* = 13, *SD* = 2.3), we analyzed our kinematic indices using linear mixed effect models (*lme*). This type of analysis has been proven to be optimal for a research like our in which the loss of data is unbalanced across experimental conditions and is often high and close to inevitable. In these conditions (if an experimental design loses balance due to missing data), *lme* was shown to suffer less severe loss of statistical power compared to mixed-model ANOVAs [121]. In particular, in order to prevent our analysis from false positives we adopted a Bayesian approach to inference based on Bayesian linear mixed effect (*blme* R package) models with an independent random intercept for every single combination of Subject, Profession type, Order version and Actor role and with the Social block and the Distance of the attendee as fixed effects [122, 123]. A comparison of models with nested fixed effects showed that none of the kinematic indices of prehensile movements we analyzed were affected by our balancing variables, therefore, data have been analyzed using *blme*, having the combination of balancing variables (Profession type, Order version and Actor role) and Subject as independent random intercept and Social block (high/low status) and Distance of the attendee from the end position (close vs. far, namely 20 vs. 120 cm) as fixed effects. In particular, the ordering of the social status block did not affected motor performance suggesting that there were no residual carryover and learning effects in our design: on average, we indeed found no significant differences, for all considered kinematic indices of prehensile movements, between the first and the second motor performance (*p*s > 0.3).

We used type 3 like two tailed *p*-values adjusting for the *F*-tests the denominator degrees-of-freedom with the Kenward-Rogers approximation implemented in KRmodcomp's function, R Package pbkrtest [124]. Among the indices that have been proposed as reliable measures of the predictive power and of the goodness of fit for Bayesian linear mixed effect models [125,126] we selected the concordance correlation coefficient, *r*_*c*_, providing a measure of the degree of agreement between the observed values and the predicted values, in the -1 to 1 range. Post-hoc tests were addressed using Welch two tailed *t*-tests and Cohen's *d* as a measure of significant effect size.

Data of the last two items of the post-experimental questionnaire have been first converted into a bipolar scale of congruency with the sign determined on the basis of the congruency between the observed and expected response. Specifically, a negative score stands for a response fully incongruent with expectations (i.e. business consultant rated as being less relevant/prestigious than the temporary worker); while a positive score stands for a response fully congruent with expectations (i.e. business consultant rated as being more relevant/prestigious than the temporary worker).

A preliminary analysis on individual responses at the post-experimental questionnaire revealed that: (1) all tested subjects correctly recalled the attendee-job association presented during the instruction phase of the experiment (100% correct responses in the first two items of the questionnaire); (2) in general tested subjects perceived the jobs associated to the attendees enrolled in the high status role as being more relevant (0.74 ± 0.19, *t* vs. 0 = 3.884, *df* = 30, *p <* 0.001), and prestigious (1.09 ± 0.23, *t* vs. 0 = 4.79, *df* = 30, *p <* 0.001) than the jobs associated to the attendees enrolled in the low status role. This was true for both the business consultant/temporary worker profession types (*M*_*relevance*_ = 0.68 ± 0.26, *t* vs. 0 = 2.51, *df* = 15, *p* = 0.02; *M*_*prestigious*_ = 1.12 ± 0.27, *t* vs. 0 = 4.14, *df* = 15, *p* = 0.001), and the entrepreneur/metalworker profession types (*M*_*relevance*_ = 0.80 ± 0.28, *t* vs. 0 = 2.86, *df* = 14, *p* = 0.012; *M*_*prestigious*_ = 1.06 ± 0.38, *t* vs. 0 = 2.78, *df* = 14, *p* = 0.014).

We considered the subjective measures extracted from these last two items as indicative of the overall effectiveness of the job manipulation but not of the individual implicit attitudes towards status difference. Given that responses at the post-experimental questionnaire were based on self-reports they were likely to reflect individual explicit (rather than implicit) attitudes towards reducing status difference from dissonance reduction or compliance with experimenter. Responses were thus likely to be biased towards null values of the scale. Furthermore on the basis of numerous studies suggesting that hand movement kinematics are useful tools for the uncovering of implicit social attitudes from explicit ones (i.e. see [127] for a review, and [128] for an example), we decided to include in the analysis all participants (although 3 of them collected a null score in at least one of the two scales).

## 3 Results

The average data on the temporal and spatial properties of the prehensile hand movement showed in Fig 3 are in strong agreement with our hypothesis that the social status of the attendee affects the speed and the accuracy of motor execution.

### 3.1 Social status affects the reach-to-grasp phase

The results from the *blme* indicated that, the main factor of the Social Status strongly affected both the amplitude (*F*_1, 1582.12_ = 22.18, *p* < 0.001, *r*_*c*_ = 0.82) and the time of peak velocity (*F*_1, 1582.26_ = 7.49, *p* = 0.006, *r*_*c*_ = 0.67) reached by the wrist before object contact. No other main effects or interaction emerged from these analyses. As depicted in Fig 3A (intervals along the segments from wrist onset to peak wrist velocity), the time the hand required to reach its maximum velocity after the movement onset increased steadily from the high (red segment) to the low (green segment) social status condition of about 4.2 *ms* (*t* = -2.1, *df* = 1611.6, *p* = 0.04, *d* = 0.11). This result was paralleled by the result on the amplitude of peak velocity (Fig 3B) which was larger in the high than the low social status condition (*M*_*High*_ = 564 *mm/s* ± 2.77 *mm/s* vs. *M*_*Low*_ = 552.3 *mm/s* ± 2.51 *mm/s*, *t* = 3.03, *df* = 1598.1, *p* = 0.002, *d* = 0.15). Notably, the direction of both these effects was consistent with Expectation 1 (and not with Expectation 2), with a faster hand movement in the high, rather than the low social status condition.

The intervals along the segments from peak wrist velocity to end wrist movement in Fig 3A depicted the deceleration phase's duration of the reach-to-grasp. This variable was affected by the Social Status (*F*_1, 1582.37_ = 3.97, *p* = 0.046, *r*_*c*_ = 0.67), not by the Distance nor by the Social Status × Distance interaction. Post hoc t-tests revealed that the deceleration phase's duration was smaller in the high (red segment: 467.25 *ms* ± 3.2 *ms*) than in the low social status condition (green segment: 474.9 *ms* ± 3.5 *ms*; t-test vs. low social status condition *t* = -1.61, *df* = 1594.9, *p* = 0.05, *d* = 0.1).

Taken together, the different temporization of hand movement kinematics across social conditions caused the overall duration of the reach-to-grasp movement (i.e. movement time) to be largely affected by the social condition of the attendee (F_2,2337.58_ = 62.4, *p* < 0.001), regardless of the Distance and the Distance × Social Status interaction, confirming Expectation 1 but not Expectation 2. According to post hoc t-tests the times spent by the hand to move from the start to the object position until the wrist stopping forward movement (Fig 3A, intervals along the segments from wrist onset to end wrist movement) were smaller in the high social status (827.64 *ms* ± 3.69 *ms*, red segment) than in the low social status condition (839.45 *ms* ± 4.06 *ms*, green segment; t-test versus high social status condition, *t* = 2.14, *df* = 1600.77, *p* = 0.03, *d* = 0.1).

### 3.2 Social status affects the lift-to-place phase

The similarity between graphs of Fig 3B and 3C suggested that the effect of the social status of the attendee on the execution of the lift-to-place phase of the movement (Fig 3C) was similar to the one observed on the reach-to-grasp phase (Fig 3B). The results of the *blme* on the amplitude of peak object's lift velocity indeed revealed a similar main effect of the Social Status (F_1,1687.141_ = 34.69, *p* < 0.001, *r*_*c*_ = 0.79). No main effects of Distance and of the Distance × Social Status interaction were observed. Post hoc t-tests revealed that the peak object's lift velocity was larger (*M*_*High*_ = 534.02 *mm/s* ± 2.17 *mm/s* vs. *M*_*Low*_ = 521.24 *mm/s* ± 2.31 *mm/s*, *t* = 4.02, *df* = 1712.0, *p <* 0.001, *d* = 0.2) and was achieved earlier in time, though not significantly (red segment in Fig 3A: 415.00 ms ± 2.51 ms vs. green segment in Fig 3A: 419.8 ms ± 2.62 ms, *t* = -1.31, *df* = 1715.1, *p* = 0.18), in the high rather than in the low social status condition (again consistent with Expectation 1).

### 3.3 Social status affects hand shaping

As depicted in Fig 3D, the social status also affected hand shaping before the contact of fingers with the target object. The output of the *blme* analysis on maximum grip aperture revealed that the Social Status (F_1,1582.62_ = 5.72, *p* = 0.016, *r*_*c*_ = 0.44) was the only significant effect. Maximum grip aperture increased significantly of 1.6147 *±* 0.6050 mm, from the high to the low social status condition (*t* = -2.00, *df* = 1195.367, *p* = 0.04, *d* = 0.10). The direction of this effect is consistent with Expectation 1 (but not with Expectation 2). The maximal grip aperture is diagnostic of the degree of carefulness/accuracy of hand shaping. It indeed reflects how planning incorporates overarching action goals into an action plan [7, 8]. Indeed, the higher the complexity of the task the more the movement will be performed with the online use of sensory feedback evolving during the action, thus producing grasp with a maximum peak grip aperture deviating more from the actual size of the target object. On this basis we were thus expecting that fast hand movements (that consistently with Expectation 1 should occur in the high social status condition) would also produce a smaller peak grip aperture than slow hand movements (that consistently with Expectation 1 should occur in the low social status condition).

## 4 Discussion

Understanding how individuals decide to control and select even simple and apparently consolidated motor responses within the social context is fundamental, as humans are inherently social beings. One strong demonstration that humans are designed to function in a social environment is provided by social facilitation. People often do better on well consolidated tasks in the presence of other people having the potential to evaluate their performance (see [70] for a meta-analysis), with such a potential being associated with the knowledge of the social status of the monitoring human [74–75]. In the present study, we demonstrate for the first time that a similar social facilitation effect can be revealed by the kinematic parameterization of a well consolidated motor act: reaching and grasping an object and translating it from one spatial location to another location. Specifically, even when not explicitly instructed, male agents acting in presence of high status attendee (but not a low status attendee) of the same gender increased the speed of execution of the act through reducing its carefulness/accuracy. When in presence of a high status attendee, relative to the case of a low status attendee, the motor act was indeed characterized by: (1) larger amplitude of peak wrist and object-lifting velocity; (2) smaller duration of the wrist deceleration phase of the movement; (3) smaller maximal grip aperture (thus deviating less from the actual size of the target object). The mere presence of an attendee with a high vs. low social status thus changes the way a consolidated prehensile hand movements is executed changing the trade-off between speed of execution and accuracy of execution [9, 103, 108].

How the attendee’s social status modulated the kinematics of a grasping-and-lifting-to-place action is further supported by the graphs of Fig 2, depicting the average velocity (Fig 2A) and grip aperture (Fig 2B) profiles in the social blocks (red and green curves for high and low social status, respectively) and in the first familiarization non-social (blue curve) phase. Curves are calculated by collapsing individual profiles over the two distance conditions (as the effect of distance was small, and in general did not change the pattern of data across social conditions).

The graphs show the effect of the social status of the attendee on the transport (Fig 2A, from t0 to t1), the grasp (Fig 2B), as well as on the lift-to-place phase (Fig 2A, from t2 to t3) of the movement. The kinematic patterns characterize well distinct qualitative spatio-temporal structures of the prehensile movements for the low (green curves) and the high (red curves) social status conditions. In Fig 2A, the different wrist velocity profiles suggest that the degree of planning of the hand movement decreased as the social status of the attendee decreased from high to low. This was signalled by the:

1. Larger peaks amplitudes reached by the red (high social status) rather than the green (low social status) curve, in both movement phases (standing for the amplitudes of peaks velocity);
2. The shorter time needed by the red (high social status) rather than the green (low social status) curve to reach its maximum after the movement onset, in both movement phases (standing for the time of peaks velocity);
3. The shorter overall time covered by the red (high social status) curve rather than the green (low social status) curve to span from the time of movement onset to the time of end movement. This means that the act of reaching, grasping, lifting and placing the object towards another person took a longer overall time when that person was appraised as a high social status individual (red), than a low social status individual (green).

These results on the temporal properties of the hand movement demonstrated that the social status of the attendee has a strong impact on the degree to which prehensile movements are planned in advance or controlled online during their actual execution. Similar results can be observed on the spatial properties of the hand shaping in general, and the grip aperture profile in particular. In Fig 2B, the maximum reached by the red (high social status) curve is smaller than the one reached by the green (low social status) curve, as if the participants needed to compute a less careful approach when in presence of the high than the low status attendee [8, 21].

Statistical results on kinematic indices of prehensile movement proficiency and observations of social prehensile movement profiles thus suggest that increasing the status of an attendee weakens the spatial accuracy demands on the transport component, and leads to a faster movement. According to models of action in which the control of motor behaviours is regulated by interdependent feedback and feedforward mechanisms [3–6], the different motor execution in presence of a high vs. low social status attendee might reflect different contribution of the planned-control components of hand transport. Specifically, our results suggest that:

1. An high social status attendee activates a motor control strategy similar to the one induced by a competitive (as compared to cooperative) social context [31]. This control strategy favours the speed over the accuracy of execution, and in turn promotes the planned-(rather than the online control-) component of hand transport movement;
2. Vice-versa, a low social status attendee activates a motor control strategy similar to the one induced by social intention, e.g. [21]. This control strategy favours the accuracy over the speed of execution, and in turn promotes the online control- (rather than the planned-) component of hand transport movement.

Our finding thus suggests that social status modulates the speed/accuracy trade-off in manual prehension. Extending such an idea the domain of social factors in general would allow reconciling our result (together with the faster hand movement induced by the gazing of conspecific found by [62]), with the seemingly different slow-down effect on hand movements produced by social intentions [21–30]. In both our experiment and the Experiment 3 of [62], the motor task was performed in the absence of any explicit requirement of social interaction between the agent and the attendee. This was different from studies investigating the effect of social intention on hand movement interaction, e.g. [21]. It is thus possible that social context affects the speed/accuracy trade-off in manual prehension depending on whether an explicit requirement of social interaction between the agent and the attendee is present/absent in the motor task, thus producing seemingly different pattern of kinematic results.

Our results show a novel type of social status effect: Action execution is systematically influenced by the way an attendee is appraised in terms of her social status. This effect is independent on the other’s intention to interact [21–30], as well as on the relative position between the agent, the object and the attendee. Following Loveland's [129] tassonomy of affordances, our study is the first to provide direct evidence that the affordances of a target object can be purely socio-culturally based (e.g. reflecting preferred interactions acquired from individual's past experience), and independent on visual information relevant for the establishment of the social context (e.g. communicative gestures). Our data indeed attest that socio-culturally selected affordances may affect motor patterning in a rather genuine way thus breathing new life into the study of the effects of social factors on cognition.

Although in our work the intentions of the other person were not expressed at all through communicative gestures, a strong effect of the social status was found. This is relevant as it provides a better understanding of the effects of the presence of others on the selection of optimal motor responses for prehensile hand movements within a social context. Our results are indeed consistent with the account of social facilitation based on social monitoring and evaluation apprehension [42, 43, 65], rather than with the account based on social arousal, originally proposed by Zajonc's [44]. The motor performance is modulated by the amount of triggering of the agent's evaluation apprehension as depending on the monitoring capacity of the attendee to evaluate the agent’s behaviour (as predicted by evaluation apprehension), rather than on the mere presence of a human attendee (as predicted by social arousal). The monitoring capacity of the attendee could have been cued by the knowledge of the attendee's social status, as individuals with high, rather than low social status, frequently endorse social roles that involve the control of others’ activities and the evaluation of others’ performance [74, 75]. In such a view, changes in motor performance that result from the presence of others depend on individual’s knowledge of whether the presence of others, in a given situation, is related to aversive or rewarding outcomes, rather than on social arousal per se. Specifically, the social status of the attendee might have affected motor performance through the induction in the agent of a state of evaluation apprehension proportional to the expected capacity of the attendee to evaluate the performed action: thus accounting for the different hand movement kinematics in the high vs. low social status condition. Notably such a difference can not be explained by a social arousal account to social facilitation assuming that mere presence of others will invariably increase an individual's physiological arousal thus improving the agent’s performance (regardless from status). Hence, our social status effect shows that the impact of social context is not limited to the agent's encoding of social intentions from visual information, as demonstrated by previous studies (see [130] for a review), but also depends on the retrieval of expectancies associated with the level of monitoring congruent with the social status of the attendee.

Such a claim is consistent with previous results. First, the lack of differences revealed by previous studies (e.g. [21, 31]) between the baseline social (i.e. acting in the presence of a human attendee who does not make explicit her willingness to interact through communicative gestures) and the non-social condition (acting in isolation or in the presence of a dummy person) is consistent with the idea that social facilitation is the product of evaluation apprehension, not of social arousal. Such a lack of a mere-presence effect could have been due to the void of an explicit definition of the attendee’s monitoring capacity, not to a null effect of social facilitation on action, e.g. [26]. Such a void of definition could have impeded the formation of a clear impression about the attendee’s capacity to evaluate/monitor the agent's performance, which is a necessary precondition for the occurrence of social facilitation driven by evaluation apprehension [66]. Secondly, the direction of our social status effect on the temporal parameters of the transport component of the hand movement (participants directed their hand to the target object and lifted it faster in the high than in the low social status condition) is consistent with the effect of eye-contact on grip kinematics found by [62]. In the Experiment 3 of [62], the attendee, depending on the gazing condition, also adopted a more or less contracted body posture: neutral in the directed gaze condition vs. contracted in the not-directed gaze condition (see left and right pictures of Fig 1 Experiment 3 in [62]). According to recent results [131, 132], the attendee's body posture could have signalled a higher social power/status of the attendee in the directed gaze condition, rather than in the not-directed gaze condition, thus producing an effect similar to the one we observed.

Our interpretation of the social status effect on the execution of prehensile hand movements is further corroborated by the way in which the distance of the attendee affected hand movement kinematics in our task. According to the motor simulation theory [133], our manipulation of the attendee's distance was likely to define different types of potential interaction between the agent and the other, while keeping constant the surveillance mode of the attendee. As a consequence, and consistently with recent evidence suggesting that an object's affordances are activated primarily when objects can be easily reached, rather than when they can not [29, 134, 135], an effect of distance would have been consistent with the idea that hand movement kinematics were moulded by social intention through motor simulation, rather than by evaluative apprehension. Since we did not observe any relevant effects of the distance of the attendee, we can conclude that in the present investigation, there was no evidence that seeing another monitoring person closer to an object evoked the object’s affordances together with the simulation of their potential interaction with the object.

However, given that no traditional explicit measures of evaluation apprehension were collected in the present study, it is possible that social status could have biased the kinematics of hand movements without influencing evaluation apprehension. However, this seems unlikely, as the behavioural effects of our social status manipulation were in-line with previously reported effects of evaluation apprehension induced by social facilitation ([66–69], see [70] for a meta-analysis). An interesting issue for further research is thus to clarify the mediator effects of variables such as evaluation apprehension, sense of reward, and/or sense of motor skillfulness.

Our finding calls for an update of the current theory of grounded cognition [136–139], which going beyond the encoding of social intention for situated action, would also consider as relevant roots for the embodied brain the individual’s knowledge of whether the presence of others in a given situation is related to aversive or rewarding outcomes. Conceiving motor behavior as a problem of Bayesian inference maximizing the utility of movement outcome given sensory, motor and task uncertainty [140] allows modeling the different motor control strategies induced by the presence of an attendee at high vs. low social status elicited by our results. These different strategies might indeed result from the agent's prior knowledge in the form of a probability distribution over possible states of the social context. Specifically, the faster the execution of the motor act, the lower would be the likelihood of receiving an aversive reward, as the speeding up of the motor act would minimize the likelihood that an attendee generating a state of evaluative apprehension (with high monitoring capacity and high social status) would detect possible unaware motor responses. This interpretation is biologically corroborated by recent evidence [30] suggesting common anatomical substrates in the ventral striatum for the encoding of both factors relevant for the processing of rewards and for the processing of social factors affecting hand movement kinematics in motor task similar to the one used in the current study.

Further studies are needed to better understand the explicit preconditions for the occurrence of our social status effects on hand-movement kinematics. Taking advantage of the evidence discussed above and the evaluation apprehension account to social facilitation, we can identify at least two strong candidate factors whose effects need to be systematically investigated irrespective of social status: (1) communicative gestures possibly implemented through body posture (e.g. expansive, open-bodied postures signalling power and dominance vs. contractive, closed-bodied posture signalling powerlessness and subordination); (2) explicit evaluation of the agent performance by the attendee (i.e. aversive vs. rewarding).

Furthermore, our choice to restrict our experimental design to the only male participants and male attendee gender congruent condition limited the external validity of our finding, although ensuring for an high internal validity (see the supplementary file S2 Document for further details). Future studies will be necessary to generalize our social status effect across genders.

In sum, to go back to our original question (see end of section 1.3) the present study demonstrates, for the first time, that when reaching, grasping and placing an object in presence of a high rather than a low status attendee, we are more prone to act favouring speed over carefulness of motor execution.

## Supporting Information

S1 Dataset Data from the experiment. Two worksheets are included in the file: (1) DATASET_reach_to_grasp, with the relevant kinematics of the reach-to-grasp phase of the movement, and (2) DATASET_lift_to_place, with the relevant kinematics of the lift-to-place phase of the movement.
(XLS)

S1 Document Pre-test for curriculum vitae selection. Short description of how the four curricula used in the Experiment have been selected experimentally.
(DOC)

S2 Document Why restricting the study to only male participants? Detailed overview of the reasons behind our choice to include only male participants in the study.
(DOC)
