# Supplementary material for: Faster but Less Careful Prehension in Presence of High, Rather than Low, Social Status Attendees
Source: PLoS One. 2016 Jun 28;11(6):e0158095. doi: 10.1371/journal.pone.0158095 (PMC4924863; doi:10.1371/journal.pone.0158095)
Supplement: S1 Document — Short description of how the four curricula used in the Experiment have been selected experimentally. (DOC) [file pone.0158095.s002.doc]

# Pre-test for curriculum vitae selection

A pretest was performed to select two pairs of curriculum vitae describing a male individual endorsing a high-status role and a low-status role. In order to do that, we first set up eight curricula: four referring to high-status roles and four referring to low-status roles. Each curriculum encompassed the first and the second name of an individual (e.g., Giacomo Faroni), the information about his educational background, his current job, hobbies and an additional-information section. For instance, in one high-status role curriculum, it was mentioned that the person graduated cum laude at the Faculty of Economics (i.e., educational background), he worked as business consultant in several firms in Milano and Trieste (i.e., current job), he liked playing tennis, horse-back riding, and travelling (i.e., hobbies). In the additional information section, it was stressed that, despite his age, he was one of the best business consultant. In one low-status role curriculum, it was mentioned that the person received the high-school diploma at the professional school, albeit he got a low mark (67/100), he was in contact with a temp agency and he had worked as home builder in the past, he liked playing football and reading comic-strips; in the additional information section, it was stressed that he had been in contact with the temp agency for ten years and his economical situation was still uncertain.

To select two pairs of curricula that differed in social status but were similar in their valence-based tone, we conducted two pretests. In the first pretest, twenty-four participants, issued from the same experimental population, were presented with the curriculum of the entrepreneur, the curriculum of the metalworker, and two additional curricula (i.e., lawyer, worked for an unemployment fund). The order of presentation of the curricula was counterbalanced across participants. Participants were asked to read and form an impression about each curriculum. Participants rated the extent to which each curriculum was prestigious and profit making (for a similar procedure, see [1, 2]) on 5-point scale, ranging from 1 (= not at all) to 5 (= very much), and then they reported their evaluation of the curriculum on a 7-point bipolar scale, ranging from -3 (= very negative) to +3 (= very positive). We selected two curricula that differ in terms of social status but were comparable in terms of valence. Specifically the curriculum of the entrepreneur and of the metalworker satisfied these criteria. Indeed, the results of the statistical analysis indicated that participants considered as more prestigious the curriculum describing the entrepreneur (*M* = 3.83 ± 0.17) than the metalworker (*M* = 2.25 ± 0.2), *t*= 6.21, *df* = 23, *p* = 0.001, *d* = 1.78. Moreover, participants judged as more profit-making the curriculum describing the entrepreneur (*M* = 3.88 ±0.18) than the metalworker (*M* = 2.04 ± 0.13), *t* = 8.24, *df*= 23, *p* = 0.001, *d* = 2.4. Finally, the curriculum pointing to the entrepreneur (*M* = 1.04 ± 0.21) was perceived as being slightly more positive than the curriculum indicating the metalworker (*M* = 0.42 ± 0.23), albeit this difference was only marginally significant, *t* = 2.01, *df*= 23, *p* = 0.06.

In the second pretest, twenty-three participants, issued from the same experimental population, were presented with the curriculum of the business consultant and of the temporary worker, and two additional curricula (i.e., notary, blue-collar worker). The order of presentation of the curricula was counterbalanced across participants. The dependent measures were the same as the one used in the previous pretest. Again, we selected two curricula that differed in terms of social status but were comparable in terms of valence. Specifically the curriculum of business consultant and of the temporary worker fitted these criteria. Indeed, results indicated that participants judged as more prestigious the curriculum describing the business consultant (*M* = 3.74 ± 0.19) than the temporary worker role (*M* = 2.52 ± 0.18), *t* = 4.04, *df =* 22, *p* = 0.001, *d* = 1.37; similarly, they considered as more profit-making the curriculum describing the business consultant (*M* = 3.52 ± 0.19) than the temporary worker (*M* = 2.35 ±0.12), *t* = 5.05, *df =* 22*, p* = 0.001, *d* = 1.54. Finally, the curriculum pointing to the business consultant (*M* = 1.04 ± 0.24) was perceived as positive as the curriculum of the temporary worker (*M* = 0.65 ± 0.21), *t* = 1.25, *df =* 22, *p* = 0.22, *d=* 0.36.

Furthermore, no difference occurred between the curriculum describing the entrepreneur and the business consultant on the prestigious (*t* = 0.37, *df*= 45, *p* = 0.71), the profit-making (*t* = 0.37, *df* = 45, *p* = .71), and the valence measure (*t* = 0.01, *df* = 45, *p* = .96). Similarly, no difference was found between the curriculum describing the metalworker and the temporary worker on the prestigious (*t* = 1.01, *df*= 45, *p* = 0.32), the profit-making (*t* = 1.75, *df*= 45, *p* = 0.09), and the valence measure (*t* = 0.74, *df*= 45, *p* = 0.46).

In sum, the two pairs of curricula included descriptions that differed in terms of prestige and income, namely in terms of social status, but were comparable in the terms of valence. Moreover, and comparing the two pretests in a between fashion analyses, high-status curricula as well as low-status curricula did not differed from each other on the social status and valence dimension.

# References

1. Fiske ST, Cuddy AJ, Glick P. Universal dimensions of social cognition: Warmth and competence. Trends Cogn Sci. 2007; 11: 77-83.
2. Brambilla M, Carnaghi A, Ravenna M. Status and cooperation shape lesbian stereotypes. Soc Psychol. 2011; 42: 101-110.
