# Supplementary material for: Faster but Less Careful Prehension in Presence of High, Rather than Low, Social Status Attendees
Source: PLoS One. 2016 Jun 28;11(6):e0158095. doi: 10.1371/journal.pone.0158095 (PMC4924863; doi:10.1371/journal.pone.0158095)
Supplement: S2 Document — Detailed overview of the reasons behind our choice to include only male participants in the study. (DOC) [file pone.0158095.s003.doc]

# Why restricting the study to only male participants?

Below we report the five major reasons lying behind our decision to restrict our study to the only male participants and male attendee gender congruent condition (not including the corresponding agent/attendee gender congruent condition with woman as agents, and the 2 corresponding agent/gender incongruent conditions.

First, several studies in the evolutionary psychology have demonstrated that the way in which males and females manifest intra-sexual competition is largely different [78] with male relying more on socio-economic status (together with physical abilities, like dominance) to promotes themselves [79, 80], while female highlighting their youth, physical attractiveness and character strength [81, 82]. According to this findings, the social status (i.e., income and prestige) used in our CVs to manipulate the perception of status was supposed to be effective for male but less for female participants.

Second, and as testified by Eurobarometer [83], men occupy to a greater extent high-status, managerial and leader occupational roles than women. This structural inequality leads perceivers to observer men more frequently in high-status jobs and women more often in low-status jobs thus shaping status expectancy for men and women accordingly, i.e., gender role theory, see [84]. Hence, and to the extent that perceivers associate men with high-status and women with low-status roles, they end up perceiving women who endorse high-status role as violating gender role stereotypes, i.e., backlash effect, see [85, 86].

Third, women who endorse high status roles are frequently penalized as violating status-hierarchy, i.e., status incongruent hypothesis, see [87]. As high status role are, at least in part, reserved to men, high-status women can be perceived as clashing the gender status prescriptions. Indeed, [87] found that women, but not men, are banned from dominant, high status displays, and they trigger harsh evaluation as threatening the status quo.

Moreover, the above mentioned results have been found to be rarely moderated by participants sex, suggesting that the gender-status bias is equally displayed by men and women, as it is also the case for implicit gender stereotype in general, e.g. [86, 88, 89].

These empirical and theoretical efforts prevent us form including in our study agent/attendee gender-congruent conditions differing for gender (both a group of male agents acting while being watched by male attendees and a group of woman agents acting while being watched by woman attendees), while using comparable CVs across gender, as high-status female attendees could have been appraised as counter-stereotypical (while high-status male attendees would have been perceived as fitting the gender stereotype), thus triggering less positive evaluation than high-status male attendees.

Fourth, several other studies have repeatedly acknowledged that both male and female individuals associated power and status more with men than women, thus corroborating the male-high status prototype [90-92]. Hence, including in our study a group composed by female agents and male attendees would likely have varied the relative status of the participants with respect to the status of the male attendee: female participants could perceive their status to be potentially lower than the one of the male attendee, independently on the explicit status manipulation we induced through the CVs.

Fifth, evidence accumulated that individuals’ cognitive processes, emotional reactions, motivations and behaviours dramatically change when embedded in an intra-group (i.e., as in our setting both the participant and the attendee share the same gender membership) or an inter-group (i.e., differently from our setting the gender membership of the participant and the attendee is different) context [93, 94]. Therefore, including in our study conditions in which the gender of the agent and the one of the attendee was incongruent might have turned the setting into a gender-based inter-group context which would have affected kinematics in a way which is beyond the scope of the present study.
